# Magnetic resonance imaging findings in central nervous system cryptococcosis: comparison between immunocompetent and immunocompromised patients

**DOI:** 10.1590/0100-3984.2016.0017

**Published:** 2017

**Authors:** Stenio Bruno Leal Duarte, Mariana Mari Oshima, João Vitor do Amaral Mesquita, Felipe Barjud Pereira do Nascimento, Paula Christina de Azevedo, Fabiano Reis

**Affiliations:** 1 MD, Resident in the Radiology Department, Faculdade de Ciências Médicas da Universidade Estadual de Campinas (FCM-Unicamp), Campinas, SP, Brazil.; 2 MD, Attending Physician, Departamento de Radiologia e Diagnóstico por Imagem, Hospital Israelita Albert Einstein, São Paulo, SP, Brazil.; 3 Master Degree, Neurologist, Head the Neuroinfectious Disease Clinic, Neurology Department, Faculdade de Ciências Médicas da Universidade Estadual de Campinas (FCM-Unicamp), Campinas, SP, Brazil.; 4 PhD, Head of the Neuroradiology Sector, Professor in the Radiology Department, Faculdade de Ciências Médicas da Universidade Estadual de Campinas (FCM-Unicamp), Campinas, SP, Brazil.

**Keywords:** Cryptococcosis/diagnostic imaging, Central nervous system infections/diagnostic imaging, Brain injuries/pathology, Magnetic resonance imaging, Diagnostic imaging, Meninges/pathology, Criptococose/diagnóstico por imagem, Infecções do sistema nervoso central/diagnóstico por imagem, Lesões encefálicas/patologia, Ressonância magnética, Diagnóstico por imagem, Meninges/patologia

## Abstract

**Objective:**

To assess the magnetic resonance imaging (MRI) patterns associated with central nervous system infection with *Cryptococcus* sp. in relation to patient immune status.

**Materials and Methods:**

This was a retrospective study of MRI data for 19 patients with neurocryptococcosis who underwent the examination between January 2000 and March 2014. The MRI characteristics examined included lesion topography, aspects of diffusion, T1-weighted images, T2-weighted images, and contrast enhancement patterns.

**Results:**

In all cases, cryptococcal infection was confirmed by cerebrospinal fluid analysis. Of the 19 patients, 10 were immunocompromised and 9 were immunocompetent. Abnormal imaging patterns occurred alone or in conjunction with other manifestations. The imaging patterns found in immunocompromised patients included the following: leptomeningeal enhancement, in 6; pachymeningeal enhancement, in 3 (due to intracranial hypotension in 2); perivascular space involvement, in 4; granulomas, in 2; hydrocephalus, in 2; miliary nodules, in 1; and plexitis, in 1. In immunocompetent patients, the following imaging patterns were observed: leptomeningeal enhancement, in 5; perivascular space involvement, in 3; granulomas, in 3; cryptococcoma, in 1; ventriculitis, in 1; and hydrocephalus, in 1. In 2 immunocompetent patients, diffusion-weighted imaging showed diffusion restriction in cerebral cryptococcal granuloma.

**Conclusion:**

In both groups, the most common imaging finding was leptomeningeal enhancement, followed by dilatation of perivascular spaces with the presence of mucoid material. Rare presentations, such as miliary nodules, plexitis, ventriculitis, and pachymeningeal enhancement, were also observed. None of the imaging patterns common to immunocompetent and immunocompromised patients differed significantly in frequency between them.

## Abstract (pt)

**Objetivo:**

Avaliar os padrões de ressonância magnética (RM) associados à infecção do sistema nervoso central por *Cryptococcus* sp. em relação ao *status* imunológico dos pacientes.

**Materiais e Métodos:**

Dados de RM de 19 casos de neurocriptococose foram analisados retrospectivamente de janeiro de 2000 a março de 2014. As características de RM examinadas incluíram: sítio da lesão, aspectos em difusão, imagens ponderadas em T1 e T2 e padrões de realce pelo contraste.

**Resultados:**

A infecção por *Cryptococcus* sp. foi confirmada pela análise do liquor em todos os casos. Dos 19 pacientes, 10 eram imunossuprimidos e 9 eram imunocompetentes. Os padrões de imagem anormais ocorreram isoladamente ou em associação com outras manifestações. Os padrões de imagem encontrados nos pacientes imunossuprimidos incluíram: realce leptomeníngeo (*n* = 6), realce paquimeníngeo (*n* = 3; 2 devidos a hipotensão intracraniana), envolvimento do espaço perivascular (*n* = 4), granulomas (*n* = 2), hidrocefalia (*n* = 2), nódulos miliares (*n* = 1) e plexite (*n* = 1). Em pacientes imunocompetentes, os padrões de imagem incluíram: realce leptomeníngeo (*n* = 5), envolvimento do espaço perivascular (*n* = 3), granulomas (*n* = 3), criptococoma (*n* = 1), ventriculite (*n* = 1) e hidrocefalia (*n* = 1). As sequências ponderadas em difusão mostraram restrição em 2 pacientes imunocompetentes com granulomas intracerebrais por criptococose.

**Conclusão:**

O achado mais comum de imagem em ambos os grupos foi realce leptomeníngeo, seguido de dilatação dos espaços perivasculares pela presença do material mucoide. Apresentações raras como nódulos miliares, plexite, ventriculite e realce paquimeníngeo foram também observadas. Nenhum dos padrões de imagem comuns aos pacientes imunocompetentes e imunossuprimidos diferiu significativamente em frequência entre eles.

## INTRODUCTION

*Cryptococcus neoformans,* a saprophytic fungus isolated from soil contaminated with bird excreta, is particularly pathogenic in immunocompromised patients^(1)^ and is the third most common pathogen in central nervous system (CNS) infections^(2)^, in patients with acquired immunodeficiency syndrome (AIDS), after infection with human immunodeficiency virus (HIV) and *Toxoplasma gondii*^(3)^. The major environmental sources of *C. neoformans* include soil contaminated with pigeon excreta (*C. neoformans* var. *neoformans* and *C. neoformans* var. *grubii*) and eucalyptus trees/decaying wood (*C. neoformans* var. *gattii*)^(3)^. *C. neoformans* var. *gattii* is found mainly in tropical and subtropical regions, whereas *C. neoformans* var. *neoformans* is encountered worldwide. *C. neoformans* var. *neoformans* usually infects immunodeficient individuals, leading to acute diffuse meningitis or meningoencephalitis. In contrast, infection with *C. neoformans* var. *gattii* more typically manifests as a granulomatous inflammatory response in immunocompetent hosts^(4)^.

The respiratory tract is the primary site of fungal infection in humans, and the yeast forms of fungi spread hematogenously from the lungs to the CNS^(5,6)^, from which they penetrate the meningeal vessel walls, migrating to the Virchow-Robin (perivascular) spaces, which subsequently become dilated following the activation of inflammatory cells and the deposition of mucoid material^(7)^. Once the fungus crosses the blood-brain barrier, the CNS provides an appropriate environment for fungal multiplication. *C. neoformans* has a predilection for the CNS because of the presence of specific neuronal substrates, especially neurotransmitters, that can be used by the fungus to produce melanin, which protects the fungus against oxidative stress, phagocytosis, and antifungal drugs, as well as modifying the host immune responses^(2)^.

The most common clinical findings in CNS cryptococcal infection are headache, nausea, and fever, less common manifestations are meningism, confusion (altered mental state), seizures, visual symptoms, and focal neurological deficit^(6,8)^. A diagnosis of fungal CNS infection must be considered in every immunocompromised patient with any of those manifestations. Cryptococcal meningitis is the leading fungal infection of the CNS in individuals with AIDS and the third leading neurological complication in HIV-infected patients^(3)^. Neurocryptococcosis had become a major concern with the spread of AIDS, and the spectrum of magnetic resonance imaging (MRI) patterns associated with CNS cryptococcal infection reflects the pathological behavior of the fungus.

The aim of this study was to examine the MRI patterns of CNS cryptococcal infection in immunocompetent and immunocompromised patients. This is of particular interest because differences have been observed between those two groups of patients in terms of the presentation of this disease and have been associated with specific virulence factors, as well as with host-pathogen interactions^(4)^.

## MATERIALS AND METHODS

We retrospectively reviewed the cranial MRI scans of 19 patients with microbiologically proven CNS cryptococcosis. We excluded patients with other associated infections (such as toxoplasmosis and tuberculosis) and those without MRI follow-up. The structural images had been acquired in 1.5 T and 3 T MRI scanners (Achieva; Philips, Best, the Netherlands). The following MRI characteristics were analyzed by an experienced neuroradiologist: lesion topography; aspects of diffusion; T1- and T2-weighted images; and contrast enhancement patterns. The images were obtained between January 2000 and March 2014. Because our study was retrospective, the image acquisition protocol was not the same for all patients. In this regard, leptomeningeal abnormalities were more conspicuous when contrast-enhanced fluid-attenuated inversion recovery (FLAIR) sequences were used.

## RESULTS

The mean age of the subjects was 41 years (range, 20-58 years); 73.7% were male, and 26.3% were female. Of the 19 patients evaluated, 10 (52.6%) were immunocompromised: 1 was a transplant recipient, and 9 had AIDS.

**Figure 1:**
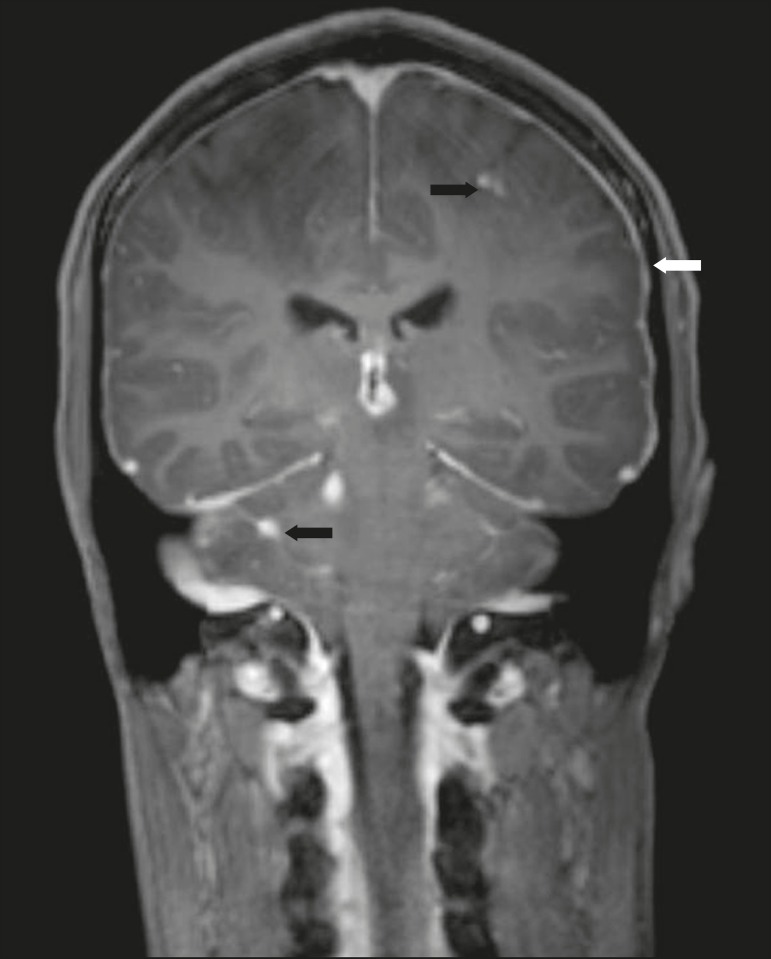
*Case 2.* Contrast-enhanced coronal T1-weighted image showing supratentorial and infratentorial focal enhancement (black arrows). In this case, there is also diffuse pachymeningeal enhancement (white arrow), due to intracranial hypotension.

**Figure 2:**
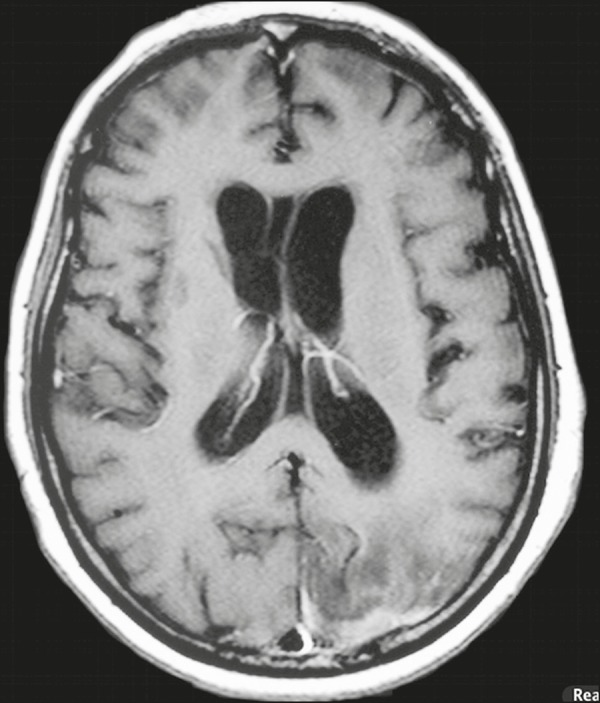
*Case 9.* Contrast-enhanced axial T1-weighted image showing mild hydrocephalus. There is also focal pachymeningeal enhancement adjacent to the left parietal lobe.

**Figure 3:**
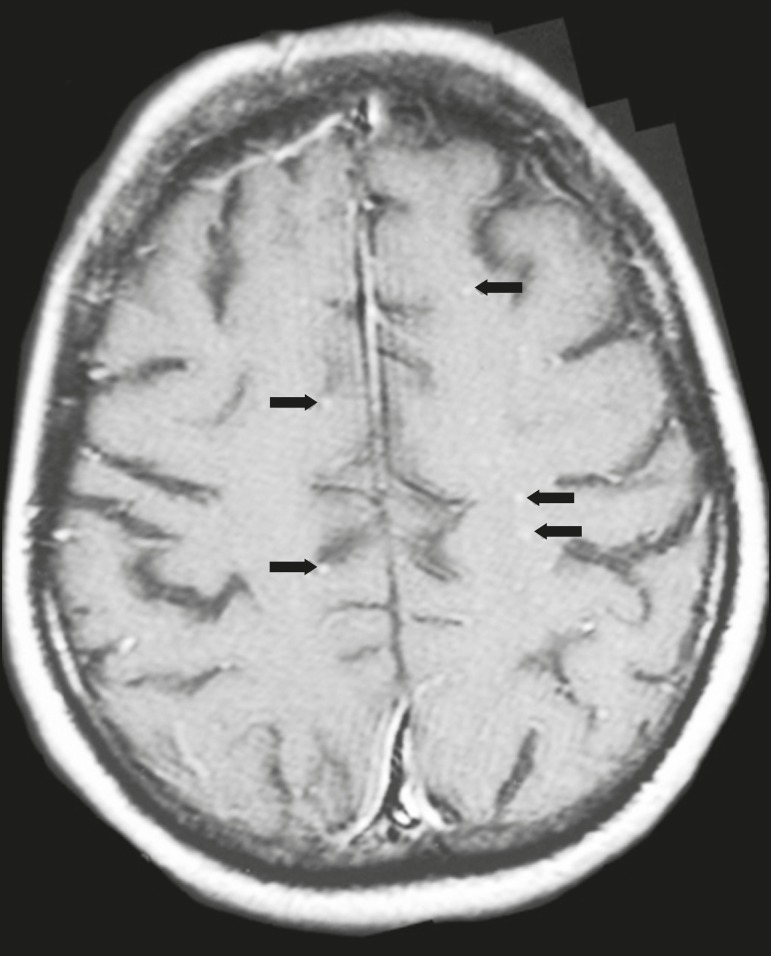
*Case 5.* Contrast-enhanced T1-weighted image showing miliary punctate enhancement at the centrum semiovale and at the cortico-subcortical junction (arrows).

**Figure 4:**
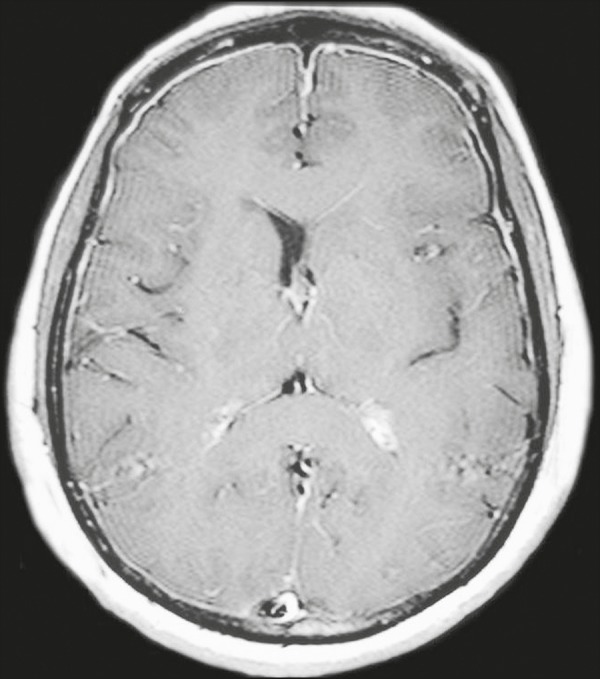
*Case 4.* Contrast-enhanced axial T1-weighted image showing bilateral choroid plexus thickening and enhancement at the ventricular atrium, mainly on the left side. In this case, pachymeningeal enhancement (due to intracranial hypotension) is also observed.

Among the 10 immunocompromised patients, the following imaging patterns were identified (Figures 1-4): leptomeningeal enhancement, in 6 (60%); pachymeningeal enhancement, in 3 (30%); perivascular space involvement, in 4 (40%); cryptococcal granulomas, in 2 (20%); hydrocephalus, in 2 (20%); miliary nodules, in 1 (10%); and plexitis, in 1 (10%). None of the immunocompromised patients showed a normal imaging pattern. Five of the patients (50%) had 2-3 concomitant MRI findings, the remaining 5 patients (50%) presenting with a single finding. Among the 3 patients with pachymeningeal enhancement, it was secondary to intracranial hypotension (with diffuse enhancement) in 2 and represented focal pachymeningeal enhancement in 1 (Table 1).

**Table 1:** Characteristics of the 19 patients with neurocryptococcosis included in the study.

Case	Age (years)	Gender	HIV status	Findings	Imaging pattern
1	55	Male	Negative/organ transplantation	Multiple	Granuloma/leptomeningeal/perivascular space
2	40	Female	Positive	Multiple	Pachymeningeal*/leptomeningeal/granuloma
3	33	Female	Positive	Single	Leptomeningeal
4	37	Female	Positive	Multiple	Leptomeningeal/pachymeningeal*/perivascular space/plexitis
5	43	Male	Positive	Single	Miliary
6	41	Male	Positive	Multiple	Leptomeningeal/hydrocephalus
7	35	Male	Negative	Single	Perivascular space
8	48	Male	Negative	Multiple	Leptomeningeal/hydrocephalus
9	44	Male	Positive	Multiple	Hydrocephalus/pachymeningeal
10	50	Male	Negative	Multiple	Perivascular space/leptomeningeal/ventriculitis
11	32	Male	Negative	Single	Granuloma
12	20	Male	Negative	Multiple	Leptomeningeal/granuloma
13	30	Male	Negative	Multiple	Perivascular space/leptomeningeal
14	58	Male	Negative	Single	Leptomeningeal
15	55	Male	Positive	Single	Perivascular space
16	29	Male	Positive	Single	Perivascular space
17	41	Male	Positive	Single	Leptomeningeal
18	26	Female	Negative	Single	Cryptococcoma
19	39	Female	Negative	Single	Granuloma

* Intracranial hypotension.

**Figure 5:**
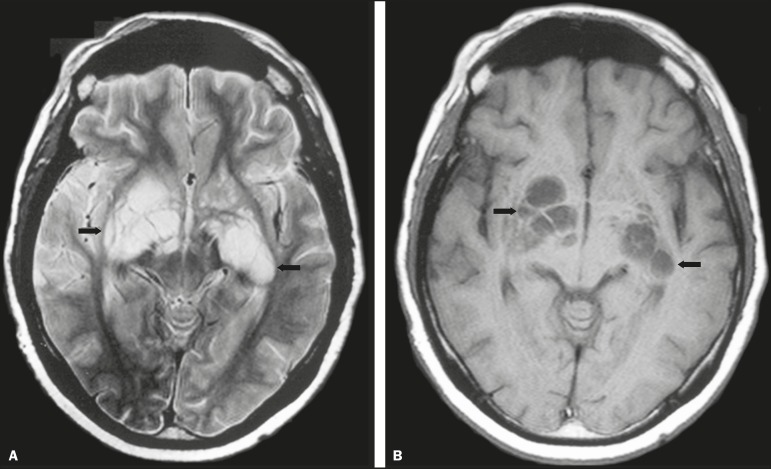
*Case 7.* Axial T2-weighted image (**A**) and axial T1-weighted image (**B**) showing bilateral dilated perivascular spaces (arrows).

**Figure 6:**
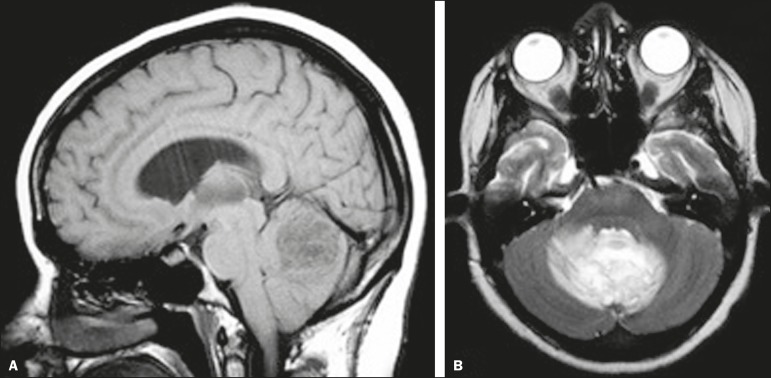
*Case 18.* Cryptococcoma. Sagittal T1-weighted image showing a lesion with a heterogeneous, hypointense signal in the superior vermis (**A**). Axial T2- weighted image of the same lesion (**B**) showing a heterogeneous hyperintense signal and perilesional edema.

**Figure 7:**
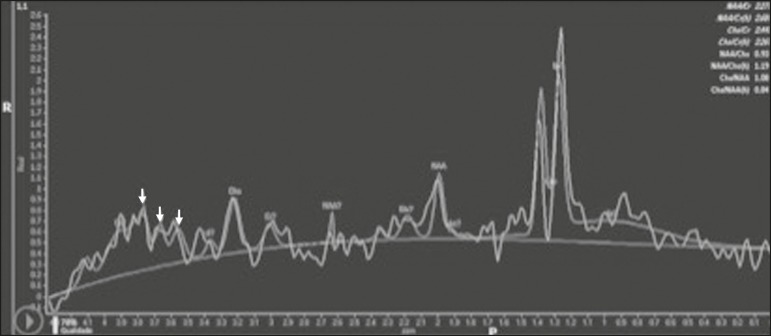
*Case 19.* Proton magnetic resonance spectroscopy of the core of the lesion tissue showing a lactate peak at 1.3 ppm and multiple signals in the region of 3.6–3.8 ppm (arrows) corresponding to trehalose.

Among the 9 immunocompetent patients, the imaging patterns identified (Figures 5-7) included the following: leptomeningeal enhancement, in 5 (55.5%); perivascular space involvement, in 3 (33.3%); cryptococcal granulomas, in 3 (33.3%); cryptococcoma, in 1 (11.1%); ventriculitis, in 1 (11.1%); and hydrocephalus, in 1 (11.1%). None of the immunocompetent patients showed a normal imaging pattern. None of the patients in this group had miliary nodules, plexitis, or pachymeningeal enhancement. Four of the patients (44.4%) had 2-3 concomitant different MRI findings, the remaining 5 patients (55.6%) presenting with a single finding (Table 1). Diffusion-weighted imaging of two immunocompetent patients showed restricted diffusion in cerebral cryptococcal granulomas. In one of our immunocompetent patients, spectroscopy showed a trehalose and lipid/lactate peak in a brain granuloma.

In the study sample as a whole, the most common imaging finding was leptomeningeal enhancement, followed by dilatation of the perivascular spaces. None of the imaging patterns common to immunocompetent and immunocompromised patients differed significantly in frequency, as assessed by Fisher's exact test.

## DISCUSSION

CNS cryptococcosis produces a wide variety of MRI features that may vary depending on the immunological status of the patient. As shown here, the MRI findings range from single to multiple alterations such as hydrocephalus, leptomeningeal/pachymeningeal enhancement, dilated perivascular spaces, miliary nodules, plexitis (via hematogenous dissemination), and pseudotumor (cryptococcoma), occurring in isolation or concomitantly with other MRI findings.

Chronic granulomatous reactions caused by *C. neoformans* are more common in immunocompetent hosts than in those with immunosuppression. On T1-weighted images, cryptococcal granulomas appear as hypointense lesions, with or without homogenous enhancement^(5)^. In the present study, the most common MRI findings in immunocompetent patients were variable-sized masses with low signal intensity on T1-weighted images and high signal intensity on T2-weighted images, accompanied by ring or nodular enhancement and vasogenic edema.

Several authors have described the radiological patterns in HIV-infected patients with CNS cryptococcosis^(2,3,6,8-12)^. The immunocompromised patients examined in the present study showed some findings that diverge from those reported in previous studies^(1,3,7,8,11)^. A granulomatous reaction, with contrast enhancement, was more common among our patients than among those evaluated in previous studies^(1,4,5,7,9,12)^. The discrepancies between our findings and those of other authors may reflect premature image acquisition after contrast administration in those other studies; low CD4 expression in the HIV-infected group in those studies; and the enhanced immunological status among our patients following the introduction of highly active antiretroviral therapy (HAART), whereas the patients evaluated in other studies had no access to HAART.

There was a time gap of 10-15 years between several important studies^(1,2,5,9)^ and the present investigation. During that period, there were major advances in image acquisition technology and clinical protocols that have markedly enhanced the diagnostic sensitivity of imaging methods. Andreula et al.^(11)^ detected leptomeningeal enhancement in 7 of 8 HIV-infected patients with CNS cryptococcosis based on an analysis of T1-weighted sequences after delayed image acquisition and the use of double the normal dose of contrast (20 mL of gadolinium, compared with the 10-mL dose used in the present study).

Dilatation of the perivascular spaces was a common finding in both groups in the present study. The perivascular space is defined as a potential space that involves a vessel and is an extension of the subarachnoid space. Most commonly located in the basal ganglia, white matter, cerebellum, and brainstem^(13)^, with a "soap bubble appearance", gelatinous round masses within the perivascular spaces appear as round foci with intermediate to low signal intensity on T1-weighted images and high signal intensity on T2-weighted images. Coalescence of the perivascular spaces is often seen, leading to a mild mass effect. Vasogenic edema is not present, and there may be little or no enhancement at the periphery of these lesions^(1-7,9,13)^.

In CNS cryptococcosis, the leptomeningeal involvement and inflammatory reaction are mild and result in the production of mucoid material within the subarachnoid space, a process that may extend to the perivascular spaces, which typically become dilated and filled with mucoid material, inflammatory cells, and organisms^(9)^. Cryptococcal meningitis is more common in the basal cisterns, although supratentorial leptomeningeal involvement was more prevalent in the study conducted by Sarkis et al.^(14)^. FLAIR sequences were assessed in two of the patients in our study. The value of contrast-enhanced T1-weighted images in detecting leptomeningeal disease is questionable, because cortical vessels can mimic meningeal enhancement, leading to erroneous radiological interpretation and misdiagnosis^(15,16)^. However, contrast-enhanced FLAIR sequences are especially useful when the data obtained with T1-weighted images are inconclusive, because blood vessels with slow blood flow do not show contrast enhancement in the latter. *In vitro* experiments^(17)^ and prospective clinical studies^(18)^ have shown that contrast-enhanced FLAIR sequences can detect superficial brain abnormalities and provide images that are more precise when compared with T1-weighted images. Consequently, contrast-enhanced FLAIR imaging should be included in the brain MRI protocol of HIV-infected patients and of patients suspected of having leptomeningeal fungal infection. Katchanov et al.^(19)^ described a pattern of leptomeningeal enhancement and vasculitis of the small perforating arteries in HIV-infected patients under immune reconstitution.

Cryptococcomas (accumulations of fungi, inflammatory cells, and gelatinous mucoid material) arise during infection and can extend to the parenchyma as focal masses, having a tumor-like appearance^(1,3,7,12)^. Again, our findings differed from data reported in the literature. For example, whereas other studies reported no enhancement in granulomas^(1,2)^, 20% of the immunocompromised patients in our study had granulomas with contrast enhancement. An extensively immunocompromised system and poor inflammatory response in the host in the pre-HAART era could partially explain these divergent findings^(19)^. Among the immunocompetent patients in our study, 44.4% had a granulomatous reaction (cryptococcoma or granuloma), a common inflammatory immune response in patients with a preserved immune system.

Intraparenchymal cryptococcomas are mass-like lesions that may mimic a brain tumor, as seen in one of our patients (case 18). The correct diagnosis of cryptococcomas, particularly in immunocompetent individuals, is challenging because these lesions may show a very high choline/N-acetylaspartate ratio in proton magnetic resonance spectroscopy^(20)^ and may require a biopsy to confirm the diagnosis (as was done here).

Choroid plexus disease (case 4) was characterized by a multilobulated cystic appearance and abnormal hyperintense signal in T2-weighted FLAIR sequences, with intense enhancement on gadolinium-enhanced T1-weighted images. Choroid plexitis is a rare manifestation of CNS cryptococcal infection^(10)^.

Restricted diffusion was observed in two cases of cerebral cryptococcal granuloma, with a solid pattern. A similar finding was reported by Kamezawa et al.^(5)^, whereas Ho et al.^(21)^ described findings contrary to this in the central cavity of a cryptococcal lesion. Consequently, diffusion cannot differentiate between fungal and bacterial infection. Cerebral infarcts, as a cause of restricted diffusion, were observed in 20% of the patients studied by Loyse et al.^(12)^. However, no cortical or lacunar infarcts were observed in our patients.

On spectroscopy, one of our patients showed a trehalose and a lipid/lactate peak. Peaks of amino acid, succinate, acetoacetate, or alanine were not found. Trehalose is specific, although not highly sensitive, for fungal infection. Using spectroscopy, Luthra et al.^(22)^ found a trehalose peak in cryptococcoma walls and cerebral mucormycosis in 5 of the 8 patients evaluated, where it appeared as multiple signals ranging from 3.6 ppm to 3.8 ppm; this profile helps to distinguish fungal infection (which shows a trehalose peak) from bacterial infection (which shows peaks in amino acid, acetoacetate, succinate, and alanine but no trehalose peak).

Fisher's exact test was used in order to compare the MRI findings between the two groups (immunocompetent and immunocompromised patients) in this study. No correlation was observed between the imaging parameters and patient immune status, in contrast with other reports^(1,6,11)^.

One limitation of the present study is the small number of patients in the sample, which could explain the lack of significant findings. None of the patients in our study had normal MRI results. That might reflect a certain bias in patient selection, because this study was conducted at a tertiary-care teaching hospital. Miliary nodules, plexitis, and pachymeningeal enhancement were found only in immunocompromised patients and at frequencies greater than those reported in the literature^(1-3,7-10)^. Pachymeningeal enhancement was diffuse in two cases (patients with other features of intracranial hypotension) and focal, indicative of focal fungal disease (not usually reported in the literature), in one.

The principal limitation of this study was its small sample size. The retrospective nature of the investigation also limited the possibilities for intervention. Further studies, involving larger numbers of patients, standardized imaging protocols, and reliable data collection with regard to the use of antiretroviral therapy and fluconazole maintenance therapy, could provide useful information on CNS cryptococcosis.
